# A Series of Cases, Exhibiting the Effects of Pressure in Cancerous and Other Diseases

**Published:** 1820-11

**Authors:** Samuel Young

**Affiliations:** Surgeon to the Cancer Institution, Gerard-street, Soho; Member of the Royal College of Surgeons, and of the London Medical and Chirurgical Society, &c.


THE LONDON

### Medical and Physical Journal.

5 OF VOL. XLIV.] NOVEMBER, 1820. [NO. 261.

"For many fortunate discoveries in medicine, and for the detection of numerous "errors, the world is indebted to the rapid circulation of Monthly Journals; "and there never existed any work to which the Faculty in EUROPE and "AMERICA were under deeper obligations than to the Medical and Physical "Journal of London, now forming a long, but an invaluable, series." RUSH.

### Original Communications, Select Observations, etc.

FOR THE LONDON MEDICAL AND PHYSICAL JOURNAL.

### *A Series of Cases, exhibiting the Effects of Pressure in Cancerous and other Diseases.* By SAMUEL YOUNG, ESQ. Surgeon to the Cancer Institution, Gerard-street, Soho; Member of the Royal College of Surgeons, and of the London Medical and Chirurgical Society, &c.

[Continued from page 273 of the last Number.]

THE following cases, important as they are in themselves, carry with them additional interest, inasmuch as they show the extension of the practice, and its successful results in other hands than my own.

One of the greatest impediments to the advance of the practice by pressure, is the great liability of its principle being perverted in application. This is not my own individual feeling alone; it has been seen and felt by several intelligent individuals, who have impartially considered the subject; by all those who have ever witnessed particularly the exhibition of the mode of the applications, and the rules and principles therein laid down. All have admitted the vital importance of such rules to the practice. All have seen and declared the perfect security and ease of the patient under the application; yet how easily they might be perverted! And, lastly, all have declared that, though they saw the important results of the practice, as well as the necessity of all the various points of its detail, yet, to their minds, convincing and simple as the principle appeared and was, yet they saw also the great difficulty there would be, under their own direction and application, to produce and keep up that efficient pressure, without detriment to the patient or otherwise, which is absolutely necessary for beneficial results.

There is, in truth, some art and a good deal of nicety required in this practice of bandaging the chest and trunk, so that all may be free, except the part where local and active pressure is made.

and kept up; and, if the little difficulties of the practice are not got over by patience and caution, by observation to correct errors, and perseverance in repeated trials, disappointments, with much inconvenience, if not injury, to the patient, will accrue; and this will do more against the practice than even the workings of malevolence itself. Such failures, indeed, are to malevolence its strength and breath: its appetite grows upon failure; and the mistakes and perversions of practice, with their consequent miseries, are the things on which it feeds.

It is particularly hard therefore with this practice, that one of the greatest sources of its evils may originate with its friends; from those who, in adopting the practice, may incautiously, though unintentionally, pervert its principle, or render it wholly nugatory, and thus afford fresh food for the hungry in slanders; while they themselves even, by these very failures, from their own insufficiency in the practice, may be added to the numbers on the list against it.

Upon such convictions it is that I have ever been so very anxious, upon all occasions that offered, to make the principles and detail of the practice *manually*, as well as theoretically, understood. It is obvious, at least to those who know the principles of the practice,—it is obvious that, without correct and efficient application, the extension of the practice may only prove an evil. To some this may appear very trifling; to those, for example, who think of nothing less than the skilful and successful extraction of stones from the human bladder, and the removal of thigh-bones from their sockets; whose minds, with an aneurismal swell of thought, ponder on nothing beneath iliacs, aortas, and carotids; who seem to think that a ligature on the arch of the great artery would have a striking effect, and that the use of the knife might even be carried to the heart itself with beneficial results.

However trifling the subject may be considered by such individuals, yet I am bold enough to affirm, and as ready to maintain, that the bandage and compress, with their various applications, involve by far the most important branches of surgery, whatever may be due to, and however justly appreciated, the higher operations, or what is termed operative surgery, may be.

The subject in question is surely as interesting as important, involving as it does the whole circle of preventive and curative treatment, as the grave and most formal dissertations which men write to, and about, one another, and their various desultory practices; accompanied with many a complimentary expletive, as "my ingenious friend," or "so ingeniously adopted," and so on: and all this, perhaps, upon the tying of an artery; whether white silk or catgut should be the material of the ligature, whether white thread or whitish-brown; whether to be cut short or long, or "*quite close*," as so "ingeniously proposed;" when, in truth, the more material object of the practice is wholly unknown or overlooked, viz. the manner of tying the knot of the ligature, and on which secondary bleedings so frequently depend.

The love of operation in all its branches, *mere sheer knife-operating*, is the great evil with our modern students.* They desert the duties of the hospital-ward for the operating-room, —the theatre, as it is called. *Theatre* indeed! where many a tragedy is performed. Here they crowd and gaze to see what they do not understand; and which, providentally, the majority of them are never called upon to perform.

It is as ridiculous as shocking to see and hear a little squeaking country lad, scarce six months old, in a London hospital, *operation-struck*, running up and down with a scalpel in his hand, in imitation of the grand carver, and talk of the capital operations and the merits of operators, whose mind is as incapable of observation as it is destitute of reflection, and knows just as much of the real spirit of his profession and the philosophy of nature as a box of basilicon. And yet such a lad, because he can let an artery play in his face, and take it up without winking, may be termed an "ingenious pupil," and, ultimately, an expert operator. So instructed, and thus accomplished, this individual of operative promise returns to his native village, where he is destined to flourish, the idol of his mother, and the wonder of the country round; and it will be well for humanity, if rabbits, cats, frogs, guinea-pigs, and dogs, are the only sufferers in the course of his professional depredations.

Let those whose capacity of observation lead them to reflect and contemplate things, follow nature in the ordinary duties of their profession: the superstructure that may be raised upon it will then have a solid basis. Then the great importance of the roller as a compress, with a right practice, will be found; and the immensity that may be done, and what wonderful effects nature is enabled to accomplish in various diseases, by and under the treatment of pressure, will then become apparent.

References have already been given to the *Further Reports*, &c. as published by Ridgway and Cox in 1818, for an ample detail of the practice; and it is intended, on some future occa-

---

* Mr. John Bell, though himself a bold and eminent operator, particularly inculcates, in his Treatise on Surgery, the necessity of attending to "the ordinary duties of the surgeon," as the most important part of the profession; and dwells particularly on the use and application of the roller. In the Manchester Infirmary, I know this practice forms a leading branch of instruction, and the pupil is regularly initiated in it. Although this is much neglected in the town-practice of some of our London hospitals, yet in others its importance is duly appreciated. At St. George's, for example, it is impossible to go over the surgery-wards without observing the great care and skill with which all the cases are treated connected with this department of the practice.

sion, to publish plates, exhibiting the various *fixed points* necessary to be made at the different courses of the roller in the application of the pressure, affording a sort of manual of the practice, and rendering the thing as minute and familiar as this sort of instruction will admit. Again however it is insisted upon, that those who, from inefficiency of application, may fail in some of their attempts, should, in common justice, be candid enough to attribute such failures to themselves, and not to the practice. Instances are constantly occurring showing this sort of abuse, by cases which were daily growing worse under an improper course of pressure, immediately and distinctly improving when a proper practice has been adopted; and also further illustrated by cases where a cure has been nearly perfected, falling back again under improper management, which have again recovered under their former right treatment, and at length ultimately been removed.

It was in the autumn of the year 1818, during a professional visit at Leweston in Dorsetshire, a seat of Mr. Robert Gordon, the Member for Cricklade, and while also the family were at Weymouth, that I had various opportunities of seeing cancerous cases, and the satisfaction of exhibiting the practice by pressure, and its mode of application in detail, to several professional gentlemen. To Mr. Gray, surgeon, at Sherborne, (from this gentleman, by the way, I have long expected some communications,) as well as to Mr. Warne and Mr. Small, of Weymouth, and others, who have all seen and felt, and expressed at the time, upon the exhibition of the practice, to the amount of the testimony already given, viz. the necessity of its detail being strictly observed; the extraordinary effects produced under it; but the difficulty, at the same time, of producing and securing that proper degree of pressure for beneficial results.

While at Weymouth and Leweston, I had also the pleasure of meeting Mr. Trowbridge, surgeon, of Cerne, the detail and success of whose cases will form so interesting and conspicuous a part of the present series They were brought to me, with the accompanying letters, by Mr. Gordon, last spring; a gentleman, it may here be proper to state, who has himself been an eye-witness of the practice, through a long, a critical, and anxious course of watching, and has borne the most unqualified and ample testimony of its benefits. On some future occasion this testimony shall be given.

(COPY.)

"Dear Sir,—I have enclosed the cases for Mr. Young, according to your request, and most ardently wish (should they be worthy of his notice for publication), they might be of service, to impress on the minds of medical men in general the necessity of their adopting his mode of treatment in cancerous complaints, if only through *humanity.*

"In all the cases I have made use of his plan, the patients have, after a day or two, expressed themselves satisfied it has lessened their sufferings. I now take this opportunity of returning you my best thanks for the many obligations I owe you, and for that of introducing me to Mr. Young; and believe me, with the greatest respect, your obliged servant, (Signed) N. I. Trowbridge."

"*Cerne; April* 20, 1820.

"Robert Gordon, Esq. M.P."

(COPY.)

"My dear Sir,—I have taken the liberty of sending you the annexed Cases, which I have treated under the method you recommend, and which, I am happy to say, has answered my expectations.

"The plan, I have every reason to believe, will produce that relief in those complaints you have so humanely intended it for. Even in the most advanced and horrid stages of the disease, I am satisfied it will procure a mitigation of pain and a considerable degree of comfort, *if the mode of applying the bandage be strictly and impartially adhered to.*

"The two other slight cases of tumor in the breasts of the female servants that came to you from the country during your visit to Weymouth, have also done well, under the applications of the adhesive plasters and bandages you advised, and which you did me the honour to entrust to my care.

"Should the communication of these cases be of utility in any way, may I beg the favour of your making the requisite alterations and amendments, as I have merely drawn them up for your own perusal.

"Believe me, dear Sir, with the greatest respect, your obedient servant, (Signed) N. I. Trowbridge."

"*Cerne; April* 20, 1820.

"To Samuel Young, Esq."

In observation to the request contained in the last paragraph of this letter, I take leave to say a few words, on venturing to publish the evidence precisely as it came to me, without one tittle of alteration. The style and manner of drawing up the cases will speak for themselves; they were sent loosely, though faithfully, as a statement of facts connected with a most important question, the author having no object of *authorship* in view; and in this way, I think, the ends of truth and justice will best be answered.

For greater accuracy, however, it may be proper to observe, that, where Mr. Trowbridge speaks of inflammation of the skin at the internal upper angle of the breast, in the first of the following cases, (the one I myself saw and treated, as stated, when at Weymouth,) it was that state of integument under cancerous progressive change *leading to ulceration*, and not what may be conveyed by the expression of mere inflammation. The whole breast was under great excitement, and the diseased and discoloured part alluded to, in a most irritable and critical state. Indeed, the patient was considered by us at the time to be in that dangerous and hopeless state which precedes the ravages of a cancerous breast, in cases where great local pain and irritation have produced constitutional disturbance to the extreme.

At the inner lower angle of the breast, where Mr. Trowbridge mentions there was much pain, evident schirrus also existed.

There is another point perhaps worthy of notice in this case, that a more accurate state of things may be conveyed, and that is, where Mr. Trowbridge mentions "a soft pulpy feel where the blue appearance was, (alluding to the integument of the upper part of the breast,) denoting the presence of *matter*." Most unquestionably this was fungus, and not matter, at least not pus, making its way through the progressively altered and diseased skin; and which was proved in the sequel, when this diseased skin gave way, and when, as seen on October 28, 1818, upon the removal of the applications, instead of matter or pus following, nothing but a dessert-spoonful of bloody discharge "issued through an opening about the size of a pea;" denoting most precisely that stage and state of the fungoid progress and nature of such and similar diseases.

Precisely the same observations may apply to the diseased and discoloured integument at the lower angle of the breast, and where fungus was also making its way; but which, to Mr. Trowbridge's touch, conveyed a fluctuating feel, which by the way such cases generally do convey when handled, though nothing but a soft fungus exists, as frequently has been proved.

These observations are made quite in a practical point of view. As to the nature of the case, no one could be more fully aware of it than Mr. Trowbridge at the time; and, during its progress whilst under his treatment, it is seen, in the following statement, that he speaks of this case when in wound, "that the whole surface had every appearance of cancer, and as if likely to commit its ravages in a short time."

Upon the subsiding of the bulk of the breast, he also states the existence of "tumor of *rocky hardness* round the nipple, and extending outwardly;" and subsequently it is also stated by this gentleman, that several cancerous filaments were removed during the progress of the treatment: circumstances sufficiently conclusive in themselves, if no other evidence existed.

The two cases which Mr. Trowbridge mentions in his letter to me, and which, in contradistinction to the following very formidable ones, he terms "slight," were incipient schirri; and no doubt, if not prevented, would ultimately have led to bad and fatal consequences. So that, from this gentleman's practice alone, we have five cases wherein the successful practice by pressure is unequivocally established.

*A Statement of Cases, as transmitted by* Mr. Trowbridge, *Surgeon, of Cerne, Dorsetshire.*

Case I.—Mrs. B—e; irregular schirrous formations, and great morbid enlargement of the whole left breast; 48 years of age, of a full habit of body, had complained of pain in the left breast for nearly sixteen years past, in consequence of a blow she had accidentally received, for which leeches and various topical applications had been had recourse to, without any evident benefit: of late it had become so extremely painful it was almost insupportable. Mr. Young being at that time at Weymouth, she was, by the desire of her medical attendant, requested to consult him; and, on Monday, October 12, 1818, he saw it, and applied two long bandages, with three compresses, over the whole breast, and wetted it with a lotion of camphorated spirit of wine and spirit of lavender equal parts. At that time it was very painful on the internal lower angle, with a large patch of inflammation on the internal upper angle, with frequent lancinating pains towards the axilla, and much tenderness down the internal part of the fore-arm. It was nearly twice the size of the other breast. Mr. Y. dressed her four or five successive days, and ordered her to take the following medicine: ℞. Hydrargy. sub. gr. iv.; pulv. antim. gr. ij.; ext. hyoscyami, gr. j.; M. f. pil. No. ij. hora somni sumend et haust. sequent cras mane. ℞. Pulv. jalapii, gr. xij.; pulv. rhei, gr. x.; tinct. sennæ, ʒ iſs. Aq. menth virid ℥ x. M. f. haust.

On the first application of the bandages for a few hours, she complained of its producing an increase of pain, with great inconvenience, but then gradually diminishing.

On Monday, October 19, she came under the care of her medical man in the country. The pain was much abated, and the inflammation somewhat lessened. Ordered the following medicine: ℞. Infus. colomb. ℥x.; tinct. ejusdem, gr. xxx.; potass. carbonat. gr. xx. M. f. haust. bis de die sumend. ℞. Pil. hydrargy. gr. iij.; pulv. antim. gr. ij. Pil. No. ij. omni alterâ nocte sumend.

*Oct.* 20.—The same as the day before. In addition to her other medicines, she was desired to take half a pint of decoction of taraxacum twice or three times in a day. Her pulse at this time was from 90 to 100; the tongue coated; with loss of appetite, and feverish heat; with dark-coloured and offensive evacuations.

*Oct.* 21.—A restless night, in more pain. A thicker compress was put over the internal lower angle, which produced relief. The inflammation still on that part; but on the upper it has put on a bluish hue, with a shining surface. Rather a more general softness in the feel of the breast. The bandage increased in tightness.

*Oct.* 22.—A more restless night, with increase of pain in the internal lower angle, which abated towards the afternoon; the general inflammation not so much to external appearance. Complaining occasionally of shooting pains in the whole inner half of the breast.

*Oct.* 23.—A better night; less pain and inflammation in the lower part, but an increase of both in the upper, with a soft pulpy feel where the blue appearance was, denoting the presence of matter; complains of soreness of the gums. The pil. hydrargyri omitted, but continued the other medicines.

*Oct.* 24.—A restless night, with shooting pains towards the axilla; in other respects, the same as on the preceding day. The alvine secretions had assumed a better colour.

*Oct.* 25.—Nearly the same as yesterday, excepting the upper part more thin, and a distinct feel of a fluid.

*Oct.* 26.—A bad night, with considerable pain throughout the whole breast, and much irritative fever. Omitted her former medicines, and took salines. On the internal lower angle is a blue cast, about the size of half-a-crown, with shooting pains. The bandage was not applied so tight. Bowels costive: took ol. ricini ℥fs. which produced four evacuations, with considerable uneasiness in the bowels.

*Oct.* 27.—Still complains of much pain on the internal half of the breast, but not so much towards the axilla; neither so much inflammation on the external part; the upper discoloured part much thinner, and a very slight crack in the skin.

*Oct.* 28.—Felt considerable pain during the whole night. On removing the applications, there was about a dessert-spoonful of bloody discharge from the internal upper part, which issued through an opening about the size of a small pea, and also excoriation to the size of half-a-crown; experiencing much shooting pain in the lower part, but the external part of the breast easy, without any darting pains towards the axilla.

*Oct.* 29.—A disturbed night, but the pain rather diminished; the discharge the same as yesterday both in colour and quantity, with the appearance of another small opening, sufficient to admit a probe, near the other. The under part of the breast thinner and softer.

*Oct.* 30.—In all respects as yesterday. The soreness of the mouth gone off. To omit the saline medicine, and have recourse to all that had been before prescribed.

*Oct.* 31.—No sleep until six in the morning, from much pain in the under part of the breast; about the same portion of discharge from the wounds as before, with a very small quantity of healthy-looking pus; another small aperture below the other two, but all within the circumference of a shilling, from whence issued a small quantity of blood, as well as from the first of the other openings. The bowels confined; therefore repeated the pil. hydrargy. sub. with the draught on the following morning.

*November* 1.—A more tranquil night, with less pain. About a table-spoonful of a sanious discharge on the removal of the dressings, which has been only dry lint; the under part appearing more to a point, as if matter was making its way through; the external part of the breast not so large nor tender, but a considerable rocky knob to be felt under the nipple, and extending outward. The medicine had the desired effect, and the evacuations mended in colour.

*Nov.* 2.—A bad night, with considerable pain, much fever, and a bloody increased discharge; the excoriated surface had come off in the dressings, sufficient to admit of two fingers being passed down towards the lower part of the breast where the point appeared the day before, and the whole surface of the wound had every appearance of cancer, and as if likely to commit its ravages in a short time. A thick small compress was applied on the internal lower part, which assisted the discharge, and produced a little ease.

*Nov.* 3.—A better night; in other respects as on yesterday. The pressure increased.

*Nov.* 4.—The same as on the day before.

*Nov.* 5.—From this time to the 26th of the same month, all things went on nearly the same; the wound appearing quite stationary, neither increasing or diminishing in size.

*Nov.* 27.—Had taken cold, which produced a troublesome cough, that caused great pain throughout the whole breast.

*Nov.* 28.—An increase of cough, with fever and ulcerated sore throat, for which she took saline and antimonial medicines, and discontinued the former medicines; the wound looking foul and rather larger, with very little discharge, and that of a very thin sanious quality.

*Nov.* 29.—The same as on the preceding day, having passed a very bad night.

*Nov.* 30.—Rather better in every respect, except the wound, which appeared the same.

*December* 1.—The same as on yesterday, and continued so until the 7th.

*Dec.* 8.—Better altogether; the wound cleaner, and looking more healthy.

*Dec.* 9, 10, 11, 12.—The same as before.

*Dec.* 13.—The surface of the wound had a smooth shining appearance, without the least disposition to heal; the pressure was in consequence increased. Complaining of loss of strength and appetite, she was desired to take decoct. cinchon. with acid sulph. dilut. Things went on nearly in this state until the beginning of February, 1819; occasionally taking the pil. hydrargy. sub. with the draught the next morning.

*Feb.* 9, 1819.—The wound had now assumed a more healthy aspect, and a few granulations had been thrown out in different parts. I forgot to mention that, during this long interval, there had been many long filamentous threads drawn out, and come away in the dressings. It had perfectly closed from the bottom of the wound; neither did it break at the lower internal angle, where there was every prospect of matter making its way through. From this time the wound very slowly improved, and did not heal until the middle of April; and to all appearance was then completely cicatrized.

On the 22d of April, whilst removing the bandage, I observed that the lint was moist: upon taking it off, the skin was abraded, which was in consequence of moving the arm briskly the evening before. It healed again in the course of a few days; and on the 12th of May the same sort of accident occurred, which also healed in a short time, and has continued perfectly sound ever since. The volume of the breast is not reduced to the size of the other; and there still remains a small floating tumor, without much pain or inconvenience, which in all probability would have been removed, had not the bandage been loosened in its tightness of application during the hot weather this summer; which is now re-applied as before, and from which she receives every comfort, saying that she cannot possibly go without it.

Case II.—Mrs. M——s, about forty years of age, of spare habit of body, naturally enjoying good health, applied for relief on the 11th March, 1819. She complained of excruciating pain in the right breast, extending into the axilla, and down the arm towards the elbow, producing considerable weakness in the whole arm. On examining the breast, a floating tumor was felt on the right side of the nipple, about the size of an egg; which she said had been there for nearly nine years, and almost constantly producing much pain, and particularly on being felt. From false delicacy, she never suffered any person to examine it; therefore no means had been used to disperse it. At length the pain became so violent, that she could no longer support it, and on the day before mentioned sent for me; when I applied the bandage with one compress, and a lotion of sp. lavender et sp. vin. camp. aa. *n. e.* Ordered her some opening medicine to keep her bowels regular.

*March* 13.—Complained that, shortly after the bandage had been applied, she had an increase of pain; which abated in the course of two hours, and felt much easier than before the bandage had been applied.

*March* 16.—Feels no pain; keeps the arm perfectly at rest; and, if possible, can perceive that the tumor is not so large.

*March* 20.—Much the same as before.

*March* 22, 26, 30.—The same.

*April* 3.—The tumor is evidently decreased in size, and feels no pain except on moving the arm, which she is particularly desired not to do.

*April* 8, 13, 18, 25.—Every thing going on gradually to a speedy recovery, and she has every hopes of being soon perfectly cured.

*April* 29.—Having incautiously moved the arm violently, had produced some slight degree of inflammation and much pain.

*May* 1.—The rules laid down were strictly attended to, in consequence of which an amendment had taken place.

*May* 7.—The breast, in its general appearance and feel, much better; the tumor had diminished in size. From this time unto the 7th of June, it had been gradually lessening, so that on that day it was not larger than a horse-bean, and is now perfectly gone. The patient has left off the bandage, but contrived something in lieu of it, as she finds a support and comfort from its application.

Case III.—Sarah Yeatman, forty-four years of age, apparently of a healthy constitution, complained of pain in the right breast about two years since, but did not perceive any tumor until six or eight months afterwards, when she applied for medical advice to a regular practitioner, without procuring any relief; and afterwards to an ignorant person who undertakes to cure cancerous complaints, without obtaining the desired effect, the disease gradually increasing in size and painfulness. On January 22, 1820, she applied to me, complaining she had felt shooting pains for many days past, denoting the formation of matter, with weakness of the arm of that side. On examination, I found a lump as large as a duck's egg on the internal part of the right breast, almost immovable, as if adhering to the ribs and edge of the sternum; with an evident fluctuation of a small quantity of fluid under the skin, and considerable extent of inflammation around; to which I recommended a bread-and-water poultice to be applied, and continued until I saw her again, which was on the 28th, as she lived many miles distant. By that time the matter had discharged itself by a small opening, scarcely sufficient to admit a probe to pass. On introducing a probe, there was a sinus about the size of a goose-quill, and about an inch in length, which I opened, and dressed with dry lint only; applying the bandage over it, using the lotion as in the foregoing cases; requesting her to come under my immediate care, which she did on February 2d, when I examined the part more minutely, and found the tumor had every characteristic feel of schirrus; the wound appearing loose and spongy, which was dressed as before, and healed in the space of eighteen days; the tumor gradually lessening in size, as well as the pain diminishing, that, at the end of ten weeks from the time I first saw her, the tumor had perfectly disappeared, and the pain gone, so that she was enabled to resume her former occupation as cook without the least inconvenience whatever.

*April* 19, 1820.

With regard to Mrs. B—e's case, (i. e. Case I.) it is now two years since I saw her in a state of hopelessness and suffering, from which, had she not been relieved by this practice of pressure,—and relieved by no other known treatment could she possibly have been, she could not have survived any length of time; or, had the application of the treatment been delayed, a few weeks must have placed the case in that situation as to render it of no avail but as a palliative.

It is now a year and a half since the final healing of the sore of this case, and this length of time has been subsequently taken before it was publicly reported, that experience might be afforded of the permanent and beneficial results of the practice: and this was the feeling of Mr. Trowbridge, as stated to me by Mr. Gordon, in not sending the case to me till so late a period as the month of April of this year, that his mind, by the lapse of time and the observation it afforded, might be fully and fairly convinced and satisfied on the subject.

The second case in the statement is sufficiently clear and decided evidence of itself, without any attendant comment or remark.

Although I have seen in some true schirri, simple suppuration, or abscess, produced before their natural growth had involved the skin in the usual way, and which has broke and healed up, as in the instance of the third case reported by Mr. Trowbridge; yet it would have been more satisfactory, if, in the statement, the quantity and appearance of the fluid discharged had been noticed, after the poulticing with bread-and-water; and, when subsequently under the treatment of pressure, whether the "loose and spongy wound," with any part of the tumor, sloughed or not; what, also, the nature of the discharge was at different periods under this last treatment; and whether, if no sloughing in masses either took place of the spongy structure of the wound or the schirrus itself, yet whether the wound had at times a sloughy appearance before it healed, and whether the discharge was offensive.

On these points, perhaps, Mr. Trowbridge will favour us with that information which will make the evidences of this case more conclusive. Such observations I have made, as worthy of the integrity I have ever observed in the correct report of cases, and to identify, if possible, their precise nature. Professional accuracy, however, whatever its acumen and precision may be, can never restrain precise diseases under precise limitations. In the features of their character, as well as their courses, however specific, they will, and do, deviate from the general definitions laid down both as to their character and progress.

In such a case as the third reported by Mr. Trowbridge, what could have been done in the common routine of poulticing, &c. had not the practice of pressure been interposed? In a practical point of view, it is immaterial whether all the formalities of the true schirrus are observed, when a loose and spongy sore is produced, with a schirrous base, which defies all the ordinary treatment of surgery to cure or suppress. I have only to add, that, through a considerable experience of many years, before the practice of pressure was adopted, I have seen several similar cases, and which have always proved fatal to the patient.

[*To be continued.*]

